# From Bench to Body: Protective *Candida*-specific Monoclonal Antibodies Show *In vivo* and Translational Potential

**DOI:** 10.33696/immunology.7.231

**Published:** 2025

**Authors:** Hong Xin

**Affiliations:** 1 Department of Microbiology, LSU Health Sciences Center, USA

**Keywords:** *Candida auris*, Monoclonal antibody (mAb), Disseminated candidiasis, Immunotherapy, Antifungal resistance

## Abstract

*Candida auris* is a multidrug-resistant fungal pathogen that presents a growing global health challenge, particularly due to its ability to cause invasive bloodstream and deep-seated infections in vulnerable patients. Monoclonal antibody (mAb)-based immunotherapy offers a novel and targeted approach to overcoming the limitations of current antifungal treatments. This commentary highlights the protective efficacy of *Candida*-specific mAbs, C3.1, 6H1, and 9F2, in *in vivo* mouse models of disseminated candidiasis. These antibodies target distinct, conserved surface antigens and significantly reduce fungal burden while improving survival outcomes. Their translational potential lies in their specificity, low toxicity, and ability to enhance host immune responses, making them strong candidates for future development as adjunct or alternative therapies for invasive fungal infections in humans.

## Introduction

The emergence of *Candida auris* as a multidrug-resistant fungal pathogen has highlighted the urgent need for effective, targeted therapies to combat systemic and bloodstream infections, particularly in immune-compromised patients. Traditional antifungal drugs, including azoles and echinocandins, are facing increasing resistance, with pan-resistant *C. auris* strains becoming more prevalent globally. Facing this challenge, monoclonal antibodies (mAbs) that recognize conserved fungal surface antigens offer promising and novel alternative strategies [1].

### Why mAbs for Candidiasis

Using the A/J mouse model, which is deficient in complement component C5 but otherwise retain relatively intact adaptive immune function, the Xin group administered mAbs prophylactically before intravenous challenge with *C. auris.* A/J mice, which lack complement component C5, are highly susceptible to systemic candidiasis due to impaired C5a-mediated neutrophil recruitment and reduced membrane attack complex formation. However, unlike models that under broad immunosuppression, A/J mice retain functional innate and adaptive immune cells, enabling the study of immune therapies in a partially compromised but responsive host. This allows for the evaluation of mAb functions such as opsonophagocytic, Fcγ receptor engagement, and immune priming. Because early *Candida* invasion involves complement evasion and neutrophil interaction, this model reflects clinically relevant conditions seen in susceptible patients, such as transient immunosuppression, reduced neutrophil recruitment, and heightened systemic inflammation. Critically, the preserved inflammatory signaling in A/J mice provides insight into how mAbs modulate fungal clearance and immune responses without the confounding effects of total immune ablation.

### Experimental Evidence and Progress

This immunological context enabled a rigorous assessment of mAb-mediated protection, beginning with C3.1, whose activity exemplifies the therapeutic potential of targeting conserved fungal glycan epitope. C3.1, an IgG3 antibody against β-1,2-mannotriose (β-Man_3_), provided the strongest protection, significantly enhancing survival and reducing fungal burden in the kidneys, brain, and heart. Notably, this protection exceeded that of micafungin, a first-line antifungal. In the original study, a single prophylactic dose of C3.1 significantly prolonged survival in A/J mice challenged with *C. auris* AR-0386. By day 35, survival reached 100% in the C3.1 group, compared to 40% in DPBS controls and 60 % in mice treated with micafungin. Kidney and brain fungal burdens were reduced from 6.6 × 10^8^ and 6.2 × 10^6^ CFUs/g in controls to 1.2 × 10^4^ and 5.0 × 10^1^ CFU s/g, respectively, with brain burdens undetectable in all C3.1-treated mice [1].

Two additional mAbs, 6H1 and 9F2 being investigated, targeted Hwp1 and Pgk1, respectively. Their effects, although less potent than C3.1, showed tissue-specific reductions in fungal burden and synergistic potential when combined [1]. In a separate experiment, 6H1 treatment led to reduced kidney fungal burden from 2.9 × 10^8^ CFUs/g in control mice to 3.8 × 10^7^ CFUs/g in treated mice. Reductions in heart and brain burdens were also observed (1.5 × 10^7^ and 6.7 × 10^5^ CFUs/g, respectively), though not all were statistically significant. When administered as a cocktail, 6H1 and 9F2 conferred complete protection, with 100% survival by day 40 compared to 20% with 6H1 alone and 0% with 9F2 alone. The cocktail also produced the most significant brain fungal burden reduction—down to 5.8 × 10^3^ CFUs/g, compared to 3.4 × 10^4^ for 6H1 and 9.3 × 10^6^ for 9F2 [1].

Key features of these mAbs, including their targets, roles in pathogenesis, and organ-specific protection, are summarized in (Table 1). Notably, C3.1 outperformed micafungin *in vivo*. Although the study did not perform formal synergy testing, C3.1-treated mice showed superior protection compared to those receiving low-dose micafungin. The observed effects suggest an independent or potentially additive mechanism that may complement antifungal therapy. The β-Man_3_ glycan epitope targeted by C3.1 is broadly expressed across multiple *Candida* species including all *C. auris* clades. Flow cytometry data demonstrate that C3.1 binds strongly to AR-0389 (Clade I) and moderately to AR-0386 (Clade IV), confirming surface accessibility across divergent geographic lineages. Additionally, our laboratory has shown that C3.1 and related mAbs inhibit growth or biofilm formation *in vitro* across multiple *C. auris* isolates, including representatives of Clades I–IV. These findings indicate that the epitopes recognized by these mAbs are structurally conserved and functionally maintained, supporting their translational value as pan-clade therapeutic candidates [1].

Mechanistically, the mAbs bind to surface-exposed fungal epitopes, confirmed through flow cytometry and confocal microscopy. The observed variation in organ-specific protection suggests that the distribution of fungal burden and epitope accessibility may differ by tissue microenvironment, highlighting the potential for localized or targeted immunotherapy. Once bound, these mAbs can recruit host effector mechanisms. C3.1, an IgG3 isotype, is particularly potent in activating the classical complement pathway [2], which enhances fungal opsonization and promotes membrane attack complex formation. In addition, all three mAbs may enhance Fcγ receptor-mediated phagocytosis by macrophages and neutrophils, facilitating intracellular fungal killing. This is especially important in models like A/J mice, where complement deficiency limits C5a signaling but leaves Fcγ receptor pathways intact. In addition to these Fc-dependent immune mechanisms, a complementary mechanism of functional synergy may arise from epitope unmasking, where the binding of one antibody alters the fungal cell wall to expose or enhance binding sites for another. This phenomenon, documented in both bacterial and fungal immunology [3,4], may explain the enhanced efficacy observed when 6H1 and 9F2 are administered in combination. Indeed, the superior protective effect of the 6H1+9F2 cocktail compared to either antibody alone supports the strategy of multi-targeted antibody therapy, analogous to combination regimens used in antiviral (e.g., HIV, COVID-19) and antibacterial treatments [5–7]. These findings not only validate the therapeutic potential of anti-*Candida* mAbs but also provide a conceptual framework for the rational design of antibody cocktails tailored to fungal pathogenesis. Protection was also observed in immunosuppressed C57BL/6 mice, indicating retained efficacy even in compromised immune contexts [1].

C3.1 demonstrated protective efficacy in both complement-deficient A/J mice and immunosuppressed C57BL/6 models. While complement activation is impaired in A/J mice, Fc-mediated phagocytosis and IgG3 binding to FcγRI likely supported fungal clearance. These findings suggest mAb protection is feasible even in partially compromised hosts, although future studies in profoundly immunodeficient models (e.g., neutropenia, IL-17RA^−^/^−^) are warranted [1–3].

Although *C. auris* can cause lethal disseminated infection, its virulence in immunocompetent mouse strains such as C57BL/6 or NE^−^/^−^ is limited, even with high-dose intravenous challenge (up to 2 × 10^8^ yeast cells), these mice showed 80–100% survival. In contrast, A/J mice, which are C5-deficient, exhibited extreme susceptibility: a 1 × 10^8^ dose caused 100% mortality within 8 days even without cyclophosphamide pretreatment. Thus, while *C. auris* is not inherently virulent in healthy hosts, mAbs like C3.1 still confer strong protection under these permissive conditions, further highlighting their therapeutic potential [8].

Furthermore, the differential tissue-specific protection, with C3.1 reducing fungal burden in the kidney and brain, 6H1 in the kidney, and 9F2 in the heart, highlight the therapeutic value of a combinatorial strategy. These patterns suggest that distinct fungal virulence factors or antigen accessibility may dominate in different organ environments. A rationally designed mAb cocktail can therefore achieve more complete systemic coverage by engaging complementary mechanisms and tissue-specific targets. This not only increases the likelihood of fungal clearance across multiple sites but also reduces the risk of therapeutic escape due to single-antigen variability. Such an approach aligns with established paradigms in antiviral and antibacterial therapy, where multi-targeted combinations enhance durability and broaden the scope of protection (Table 2).

### Translational Potential and Future Directions

Beyond systemic protection, these findings have important translational implications for topical and mucosal applications, particularly in the prevention of colonization and biofilm-associated infections. *C. auris* is notorious for persisting on skin, hospital surfaces, and medical devices, contributing to nosocomial outbreaks and invasive infections in high-risk patients. Surface-binding mAbs, such as C3.1 and 9F2, could be adapted for use in topical formulations, wound dressings, catheter coatings, or mucosal sprays to prevent fungal attachment and colonization at vulnerable anatomical sites. This approach is particularly promising in settings where systemic antifungal drugs are either poorly tolerated or ineffective, such as in patients with renal insufficiency, neonates, or those requiring long-term catheterization. Traditional antifungal agents often fail to penetrate established fungal biofilms on medical devices, but antibodies targeting surface-exposed fungal antigens may reach these structures more effectively and interfere with adhesin or matrix stability. Indeed, subsequent findings by the Xin group demonstrated that C3.1 and 9F2 inhibit biofilm formation, further supporting their utility in preventing device-associated fungal infections [14].

Notably, antibodies like 9F2, which target moonlighting glycolytic enzymes such as Pgk1 [15,16], may not only mark fungi for immune clearance but also directly impair fungal metabolism or viability. This dual mechanism—both immune-mediated and direct inhibitory—reflects an emerging class of antimicrobial antibodies that go beyond passive targeting. Such mAbs may disrupt key fungal survival pathways, particularly under nutrient-limited or biofilm-associated conditions, where metabolic enzymes are upregulated and exposed on the cell surface. Together, these properties position anti-*Candida* mAbs as versatile tools for both therapeutic intervention and infection prevention, with the potential to bridge the gap between systemic and localized antifungal strategies in clinical care.

This study underscores the potential of mAbs to serve not only as immunological tools for dissecting host-pathogen interactions but also as viable therapeutic candidates for clinical application. Their high specificity allows for targeted engagement of fungal epitopes with minimal off-target effects, while their favorable safety profile and lack of inherent toxicity make them especially beneficial for immunocompromised patient populations. Moreover, unlike traditional antifungal agents, mAbs do not impose direct selective pressure on fungal metabolic pathways, thereby reducing the likelihood of resistance development and offering a sustainable therapeutic modality. Furthermore, the scalability of mAb production under current good manufacturing practice (cGMP) conditions aligns well with clinical translation goals, supporting the feasibility of rapid deployment in outbreak or high-risk scenarios. To optimize clinical efficacy, future studies must evaluate the relative advantages of therapeutic versus prophylactic administration, especially in patient populations with various levels of immune competence. Moreover, a detailed investigation of Fc-receptor interactions and downstream effector pathways will be essential for refining antibody formats and maximizing *in vivo* efficacy. Additional preclinical testing in mucosal, skin, or catheter-associated infection models will further validate real-world applications. Finally, humanization of lead candidates and pharmacokinetic and pharmacodynamic profiling are necessary milestones toward regulatory approval and clinical readiness.

### Limitations and Challenges

Limitations of the current study include limited testing in profoundly immunocompromised models, lack of pharmacokinetic or toxicity data, and need for humanized versions of the mAbs. Additionally, the mechanism of synergistic protection from mAb cocktails requires further elucidation, including whether epitope unmasking or direct antifungal activity plays a key role.

### Way Forward

Future work should focus on humanization of lead mAbs, formulation optimization, and testing in humanized and neutropenic models. Clinical translation will require scalable production and PK/PD evaluation. Additionally, combination strategies with antifungals or vaccine platforms could broaden the therapeutic impact of antibody-based approaches.

## Conclusion

The work of Xin group lays a foundation for the next generation of antibody-based therapies targeting fungal pathogens. By demonstrating *in vivo* protection using mAbs that target conserved and functionally relevant cell surface antigens on *Candida auris*, this study bridges the gap between experimental immunology and translational application. Moreover, the synergistic activity of antibody combinations opens the door to rational cocktail design, which may mitigate resistance and increase therapeutic durability. As antimicrobial resistance continues to rise, particularly among opportunistic fungal pathogens, antibody-based immunotherapies represent a timely and innovative strategy. This commentary highlights a promising therapeutic strategy and underscores the critical need to expand and diversify antifungal modalities in response to the growing threat of multidrug-resistant fungal pathogens.

## Figures and Tables

**Table 1.** Characteristics of protective monoclonal antibodies evaluated against *Candida auris*.

mAb	Isotype	Target	Role in Pathogenesis	Protection Observed
C3.1	IgG3	β-Man3	Cell wall glycan, conserved	Kidney, brain
6H1	IgG2b	Hwp1	Adhesion, biofilm formation	Kidney
9F2	IgG2a	Pgk1	Moonlighting glycolytic protein	Heart

**Table 2.** Broader summary of mAbs evaluated or approved for fungal, viral, and bacterial infectious diseases.

Antibody	Target	Pathogen	Disease/Model	Stage	Mechanism	Ref.
C3.1	*β*-Man3 glycan	*Candida* spp.	Disseminated candidiasis	Preclinical	Fungal clearance via complement and *Fcγ* opsonization	[1]
6H1	Hwp1	*Candida albicans*	Biofilm/adhesion models	Preclinical	Blocks adhesion and hyphal biofilm	[1]
9F2	Pgk1	*Candida* spp.	Cardiac dissemination	Preclinical	Inhibit growth, inhibit biofilm formation, possible direct killing	[1]
Bezlotoxumab	*Clostridium difficile* toxin B	*Clostridioides difficile*	Recurrent CDI	Approved	Neutralizes TcdB toxin	[9]
Inmazeb	Ebola GP (triple mAb cocktail)	Ebolavirus	Ebola virus disease	Approved	Neutralizes GP entry function	[10]
Shigamab	Shiga-like toxin 1/2	EHEC (*E. coli*)	Hemolytic uremic syndrome	Clinical	Neutralizes shiga toxin	[11]
MEDI3902	PcrV/PA	*Pseudomonas aeruginosa*	Ventilator-associated pneumonia	Clinical	Blocks type III secretion system	[12]
Suvratoxumab	α-toxin	*Staphylococcus aureus*	*S. aureus* pneumonia	Phase 2	Neutralizes α-toxin	[13]
